# Effect of ultrasound-guided small needle knife on patients with knee osteoarthritis: a randomized clinical trial

**DOI:** 10.3389/fmed.2026.1902456

**Published:** 2026-09-04

**Authors:** Xuefei Li, Lei Duan, Zhijun Hu, Bing Xu, Gengqi Wang

**Affiliations:** 1 Department of Traumatic Orthopedics, Yueyang Integrated Traditional Chinese and Western Medicine Hospital Affiliated to Shanghai University of Traditional Chinese Medicine, Shanghai, China; 2 Department of Rehabilitation Medicine, Longhua Hospital Affiliated to Shanghai University of Traditional Chinese Medicine, Shanghai, China

**Keywords:** complementary and alternative medicine, knee osteoarthritis, randomized clinical trial, small needle knife, ultrasound-guided

## Abstract

**Background:**

Although ultrasound-guided small needle knife (UG–SNK) treatment combined with resistance exercise has shown benefits for knee osteoarthritis (OA) patients, evidence supporting this combination remains limited.

**Objective:**

To evaluate the efficacy and safety of combining ultrasound–guided small needle knife treatment with resistance exercise for patients with knee osteoarthritis.

**Methods:**

(1) *Design, Setting, and Participants* This was an 8-week open-label, analyst-blinded, randomized clinical trial conducted from May 2024 to October 2024. A total of 80 participants with knee osteoarthritis were recruited and randomly assigned to two groups on average: the ultrasound–guided small needle knife combined with resistance exercise (UG-SNK-RE) and the nonsteroidal anti-inflammatory drug combined with resistance exercise (NSAIDs-RE). (2) *Interventions* Both groups participated in resistance exercises twice daily for 4 weeks, completing 56 sessions. The NSAID group was instructed to take celecoxib (200 mg/day) as directed. The UG-SNK-RE group received ultrasound-guided small needle knife treatment once a week for 4 weeks, totaling 4 sessions. (3) *Main Outcomes and Measures* The primary outcome was the change in Visual Analog Scale (VAS) scores from baseline to weeks 4 and 8. Secondary outcomes included the Western Ontario and McMaster Universities Osteoarthritis Index (WOMAC) and ultrasound measurements (patellar bursa effusion depth, synovial thickness, and thickness of the rectus femoris, vastus lateralis, vastus medialis oblique, and medial and lateral collateral ligaments).

**Results:**

A total of 80 participants (mean [SD] age, 61.49 [10.5] years; 42 men [52.5%]) were included. At week 4, the UG-SNK-RE group showed a significant improvement in VAS scores, with a mean difference of −2.5 (−2.86 to −2.14). At week 4, the VAS score difference between the UG-SNK-RE and NSAIDs-RE groups was −1.0 (−1.40 to −0.61; *P* < 0.001). Secondary outcomes also showed significant differences. The effects of UG-SNK combined with resistance exercise persisted at the 8-week follow-up.

**Conclusion:**

In this randomized clinical trial, ultrasound-guided small needle knife combined with resistance exercise was more effective than NSAIDs combined with resistance exercise in improving pain, functional recovery, and muscle condition at both week 4 and week 8. These findings suggest that ultrasound-guided small needle knife treatment combined with resistance exercise is a viable alternative for managing knee osteoarthritis.

**Trial registration:**

International Traditional Medicine Clinical Trial Registry: ITMCTR2024000182.

## Introduction

Osteoarthritis (OA) is a common degenerative disease, with knee osteoarthritis (KOA) being the most prevalent form (1). KOA is primarily characterized by chronic joint pain and reduced mobility, which lead to significant healthcare costs and a heavy burden on medical insurance due to its widespread occurrence (2, 3). The condition is associated with various pathological changes, including cartilage degeneration, synovial hyperplasia, and diminished muscle mass and strength (4–7). Currently, the clinical management of KOA adopts a hierarchical and comprehensive framework covering both conservative and surgical therapeutic strategies, which are tailored according to disease severity, patient age, and functional status.

Conservative interventions serve as the first-line treatment for early and moderate KOA, encompassing multiple standardized modalities including pharmacological therapy, physical agent therapy, structured exercise rehabilitation, and intra-articular injectable treatments (8). Although nonsteroidal anti-inflammatory drugs (NSAIDs) remain the cornerstone of treatment for OA (9, 10), their potential side effects—such as cardiovascular, gastrointestinal, and renal complications—are important considerations (11, 12). Beyond oral medications, topical analgesics and intra-articular injections of corticosteroids or hyaluronic acid are widely applied alternative pharmacological approaches, which achieve targeted anti-inflammatory and analgesic effects with reduced systemic adverse reactions (8).

Nonpharmacological conservative strategies, including resistance training, aerobic exercise, and physical therapy modalities such as high-intensity laser and ultrasonic therapy, have been well validated to improve joint function, enhance periarticular muscle strength, and relieve KOA symptoms (13–15). Nevertheless, the prolonged treatment cycle and rigorous adherence requirements of these physical and exercise interventions often result in low patient compliance, restricting their long-term therapeutic efficacy (13, 14). For advanced KOA with irreversible joint structural damage and intractable symptoms that fail to respond to standardized conservative management, surgical interventions including arthroscopic debridement, osteotomy, and total knee arthroplasty are routinely implemented to reconstruct joint function and alleviate severe disability (16). Given the inherent limitations of conventional pharmacological and nonpharmacological conservative treatments, as well as the invasive nature and high economic cost of surgical procedures, individuals with KOA frequently seek complementary and alternative medical options to optimize disease management and improve long-term quality of life (17).

The small needle knife is an alternative therapeutic tool that resembles an acupuncture needle but has a flat-edged tip. It has been widely used in treating musculoskeletal disorders (18–20) and may offer an effective approach to managing the symptoms of knee osteoarthritis (KOA). A clinical trial demonstrated that the small needle knife could improve knee joint mechanical balance, reduce pain, and enhance functional recovery (21). The mechanism by which it alleviates OA pain is thought to involve the inhibition of the NLRP3 inflammasome and interleukin-1β (IL-1β) expression in the central hippocampus, a reduction in substance P (SP) levels in the dorsal root ganglion (DRG), a decrease in muscle tone, and improvement in tissue ischemia and hypoxia (22–24). Recent clinical studies have sought to evaluate the safety and efficacy of small needle knife therapy for KOA patients (25, 26). One trial comparing the small needle knife with oral NSAIDs found that the small needle knife group showed significantly greater improvements in both the Oxford Knee Score and gait rhythm (25). However, the study had limitations related to safety and objectivity.

Therefore, the effectiveness, objective outcomes, and safety of the small needle knife in clinical practice for treating KOA remain unclear. This study aims to evaluate the efficacy and safety of ultrasound-guided small needle knife treatment combined with resistance exercise, using both objective measures and subjective assessments, to alleviate knee OA pain in middle-aged and elderly individuals.

## Methods

### Study design

This randomized controlled trial (RCT) included two groups with blinded evaluators. Participants who met the inclusion and exclusion criteria were randomly assigned to either the ultrasound-guided small needle knife combined with resistance exercise (UG-SNK-RE, intervention group) or the oral nonsteroidal anti-inflammatory drug combined with resistance exercise (NSAIDs-RE, control group). Detailed information on the trial protocol and statistical analysis methods can be found in Supplementary Material 1 (protocol). The study was approved by the Ethics Committee of Longhua Hospital, affiliated with Shanghai University of Traditional Chinese Medicine (approval number: 2024LCSY062), and all participants provided written informed consent. The trial adhered to the Consolidated Standards of Reporting Trials (CONSORT) guidelines (27).

### Participants

Participants were recruited from Longhua Hospital, affiliated with Shanghai University of Traditional Chinese Medicine, between May and October 2024. Inclusion criteria were: (1) age between 45 and 70 years, (2) clinical symptoms of knee osteoarthritis (KOA), defined as recurrent knee pain lasting for more than 6 months or persistent knee pain in the past month (28), (3) a Visual Analog Scale (VAS) score of ≥3 (0–10 scale, where 0 indicates no pain) (29), and (4) radiological evidence of KOA based on the Kellgren−Lawrence grading system, including grades I−III (30). Exclusion criteria included: (1) uncontrolled joint infection, active joint tuberculosis, acute knee arthritis, rheumatoid arthritis, or local wounds, (2) meniscus or cruciate ligament injury, (3) severe knee deformity, or significant vascular, neurological, or osteoporotic conditions, (4) recent use (within the past 6 months) of medications affecting bone metabolism, such as estrogen, steroids, calcitonin, parathyroid hormone, bisphosphonates, vitamin D, anticonvulsants, diuretics, and others, (5) severe comorbidities, including primary type I diseases such as cardiovascular, cerebrovascular, or hematopoietic system disorders (31), (6) severe liver or kidney dysfunction, psychiatric conditions, or dementia, (7) allergies, or (8) active gastrointestinal conditions, such as bleeding, ulcers, or perforation (32).

### Intervention

Both groups of participants received two hours of health education prior to enrollment to enhance compliance. The UG-SNK-RE intervention protocol involved using a small needle knife, guided by ultrasound, to release the patellar medial and lateral retinaculum, as well as the starting and ending points of the medial and lateral collateral ligaments, quadriceps tendon, and tender points (33). This procedure was combined with resistance exercises. The ultrasound-guided small needle knife treatments were performed once a week by experienced practitioners (HZJ, President of the Shanghai Needle Knife Society). The NSAIDs-RE regimen consisted of daily oral administration of 200 mg of celecoxib and resistance exercises. Participants in both groups engaged in resistance exercises twice daily, the resistance-training protocol was as follows: a 2-kg sandbag was fastened to the affected limb. Each training set contained four movements: Supine resistance straight leg raise, lateral resistance straight leg raise, sitting resistance straight leg raise and standing resistance knee bend. Each exercise was performed 20 repetitions, with two sets completed per day. The detailed exercise protocols provided in Supplementary Material 2. To support adherence, follow-up telephone calls were made twice a week to remind participants about their exercise routine and address any questions. The principal investigator oversaw the trial to ensure compliance with the clinical trial protocol.

### Randomization and blinding

A data administrator, who was not involved in the clinical trial, prepared a randomization list with a 1:1 allocation ratio. To ensure allocation concealment, random block sizes of 4, 6, and 8 were used. Treatment allocations were placed in sequentially numbered, opaque, sealed envelopes, which were opened only after participants who met the inclusion and exclusion criteria completed the baseline assessment. Data analysts conducted blinding of the treatment groups during the analysis phase.

### Outcomes

Outcomes were measured at baseline, after 4 weeks of treatment, and at the 8-week follow-up using paper questionnaires. The primary outcome was the VAS score, which ranges from 0 to 10 (0: no pain; 10: maximum pain) (34).

Secondary outcomes included the WOMAC score, ultrasound indicators, and electromyographic measurements. The WOMAC index assesses osteoarthritis severity, evaluating pain (5 questions), stiffness (2 questions), and functional status (17 questions) (35, 36). Each item is rated from 0 to 4, with higher scores indicating worse symptoms.

Ultrasound indicators measured included patellar bursa effusion depth, synovial thickness, and the thickness of the rectus femoris, vastus lateralis, vastus medialis oblique, and the medial and lateral collateral ligaments (37, 38). Ultrasound assessments were performed at baseline, 4 weeks, and 8 weeks of follow-up using an ultrasound device (manufactured by Siemens) and a 10–15MHz high-frequency probe. Researchers instructed participants to sit for scans of the anterior, posterior, medial, and lateral aspects of the knee joint. These scans evaluated patellar effusion depth, synovial thickness after knee flexion, and collateral ligament thickness after knee extension. A suprapatellar sac fluid volume ≥ 2 mm was considered indicative of fluid accumulation (39), while synovial thickness ≥ 2 mm was classified as synovial hyperplasia (40). Thicker medial and lateral collateral ligaments were associated with more severe joint damage (41). Muscle thickness was measured in a supine position using high-frequency probes, assessing the rectus femoris, vastus lateralis, and vastus medialis oblique (42, 43).

### Statistical analysis

The sample size was calculated using PASS software. Based on previous research (44), the standard deviation (SD) between the two groups was assumed to be 2.5. The estimated change in the VAS score for knee pain after intervention was 2.0, which is considered clinically significant (45). To achieve a two-sided test with 90% power, a 5% significance level (alpha error), and an anticipated dropout rate of 20%, a total of 80 participants (40 per group) was required (46).

Statistical analyses were performed on an intention-to-treat basis. Changes in the primary outcome (VAS) and secondary outcomes were compared using a mixed-effects model that accounted for the interaction between time and group. Bonferroni correction was applied to adjust for multiple comparisons. Baseline characteristics for each group were presented as mean (SD). Clinical significance was defined by the proportion of participants who experienced a reduction of at least 2 points from baseline in the VAS score. A two-sided test was conducted on all available data, with a *P* value < 0.05 considered statistically significant. All analyses were performed using SPSS 25.0 software by statisticians who were blinded to group allocation.

## Results

### Participant characteristics

**Figure 1:**
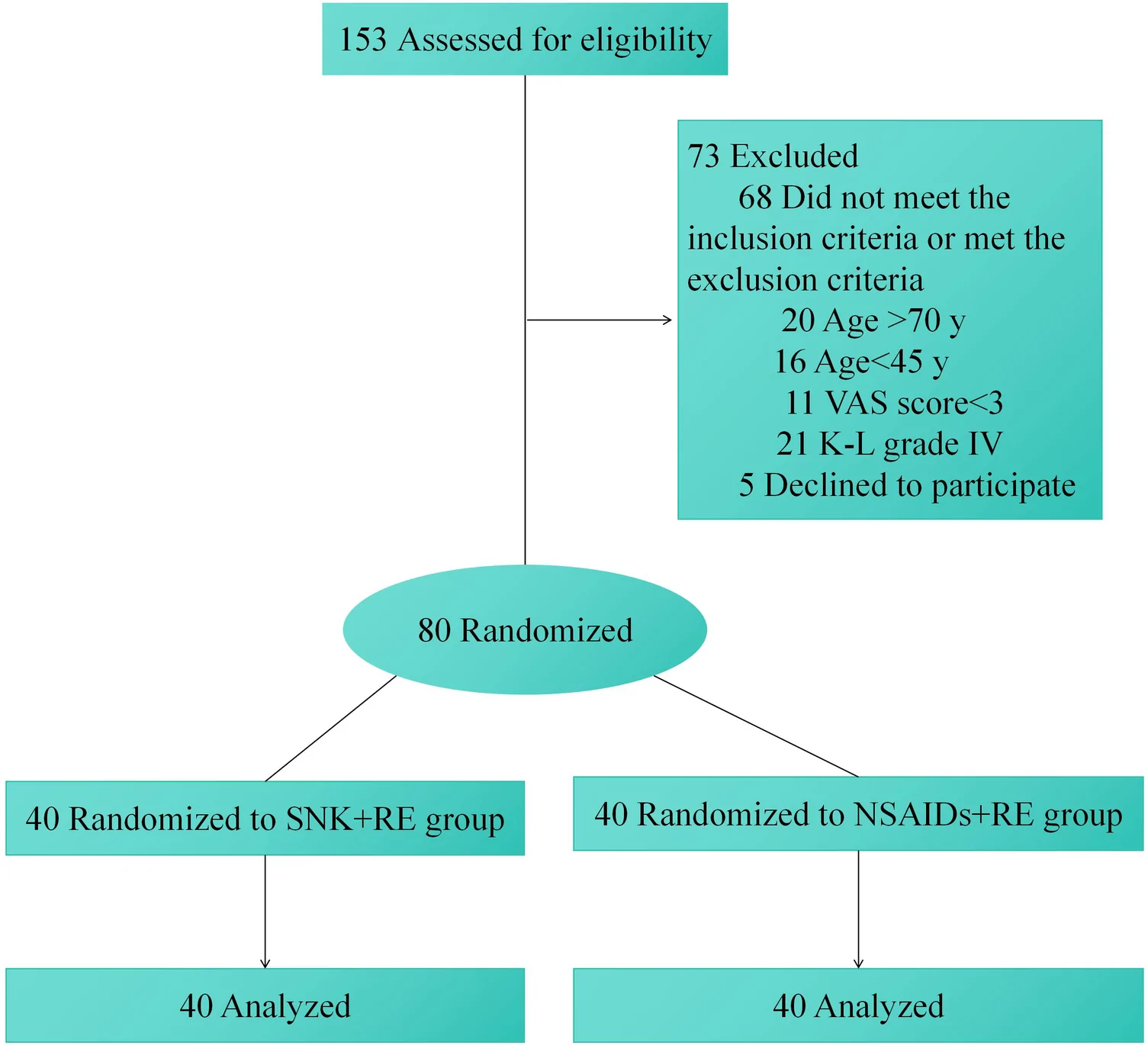
Study flow diagram.

A total of 80 participants with knee osteoarthritis (mean [SD] age, 61.49 [10.5] years; 42 men [52.5%]) who met the inclusion and exclusion criteria were randomly assigned to either the experimental group (ultrasound-guided small needle knife combined with resistance exercise, UG-SNK-RE, *n* = 40) or the control group (NSAIDs combined with resistance exercise, NSAIDs-RE, *n* = 40) (Table 1). All 80 participants completed the measurements at the designated time points (Figure 1).

**Table 1:** Baseline characteristics of the participants.

Characteristic	UG-SNK-RE (*n* = 40)	NSAIDs-RE (*n* = 40)
Age, mean (SD), years	61.1 (6.6)	61.9 (7.1)
Sex		
Male	21 (52.5)	21 (52.5)
Female	19 (47.5)	19 (47.5)
Height, mean (SD), cm	167.5 (7.6)	168.9 (8.5)
Weight, mean (SD), Kg	56.8 (8.8)	59.3 (9.3)
VAS score,mean (SD)	5.2 (1.0)	5.1 (1.0)
WOMAC pain, mean (SD)	10.1 (2.6)	10.0 (2.0)
WOMAC stiffness, mean (SD)	5.0 (1.3)	4.9 (1.2)
WOMAC function, mean (SD)	25.1 (4.4)	25.2 (4.8)
Depth of patellar sac effusion, mean (SD), mm	3.6 (0.9)	3.6 (1.0)
synovial thickness, mean (SD), mm	3.2 (1.1)	3.2 (1.2)
Thickness of rectus femoris, mean (SD), mm	13.0 (1.6)	12.9 (1.5)
Thickness of vastus lateralis, mean (SD), mm	17.0 (1.0)	16.8 (1.3)
Thickness of vastus medialis oblique, mean (SD), mm	10.9 (1.2)	11.0 (1.2)
Thickness of medial collateral ligament, mean (SD), mm	5.4 (0.9)	5.4 (1.0)
Thickness of lateral collateral ligament, mean (SD), mm	5.0 (0.5)	4.9 (0.7)

### Efficacy

**Figure 2:**
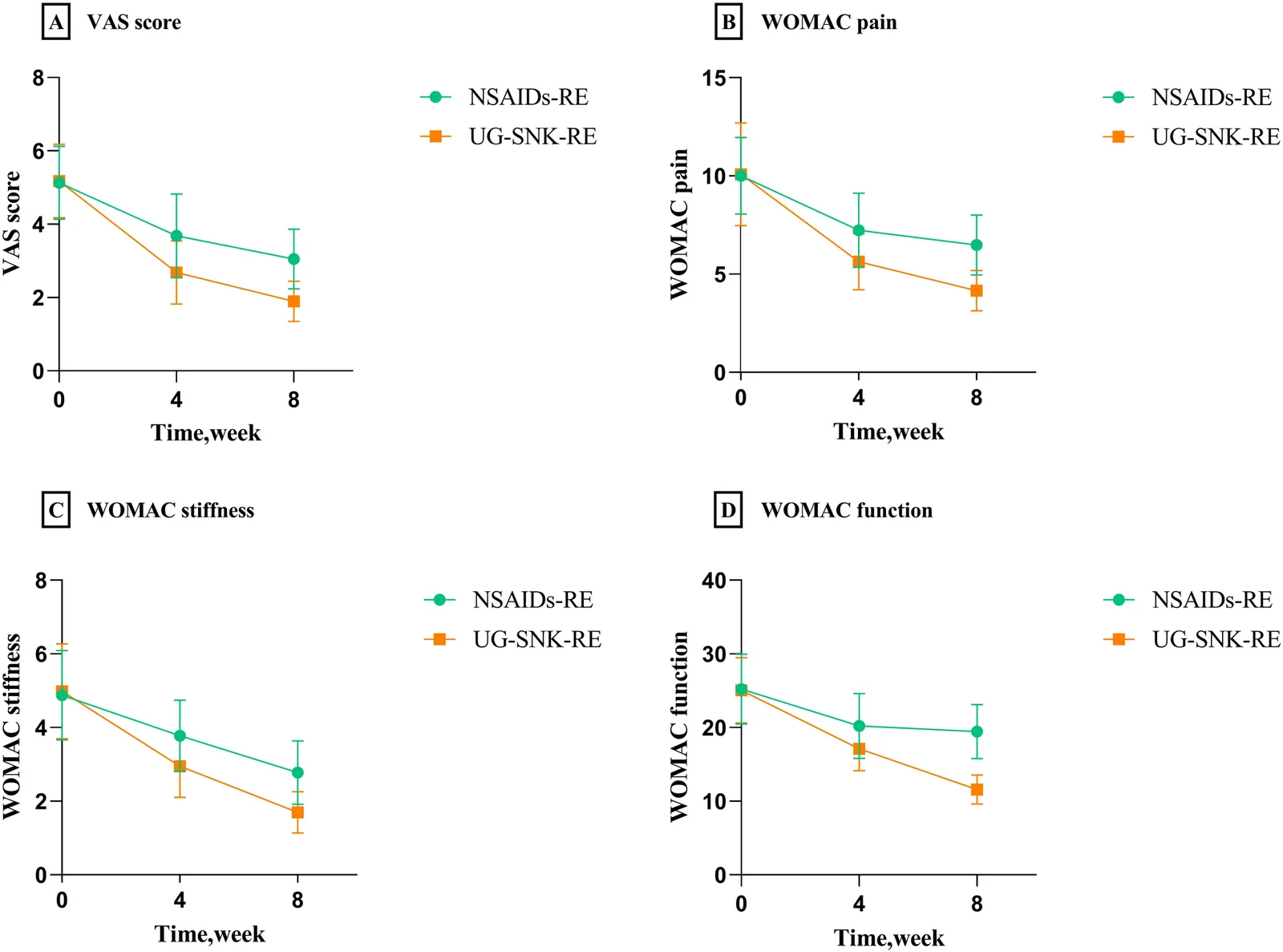
Changes in outcomes among groups over time. Sub-panels: (A) VAS score; (B) WOMAC pain; (C) WOMAC stiffness; (D) WOMAC function. The VAS score is an 11-point scale, with 0 indicating no pain and 10 indicating the worst pain. The WOMAC index assesses osteoarthritis severity, evaluating pain, stiffness, and functional status. WOMAC pain includes 5 questions, WOMAC stiffness includes 2 questions, WOMAC function includes 17 questions. Each question is rated from 0 to 4, with higher scores indicating worse symptoms. 0 indicates before intervention; 4 indicates immediately after treatment for 4 weeks; 8 indicates follow-up for 4 weeks.

The differences in VAS scores between the UG-SNK-RE and NSAIDs-RE groups at the study time points were analyzed using a linear mixed model (LMM). The results showed changes in VAS scores from baseline (week 0), post-treatment (week 4), and the 8-week follow-up (week 8). Compared with the NSAIDs-RE group, the UG-SNK-RE group experienced a significantly greater reduction in VAS scores at all time points. At week 4, the NSAIDs-RE group showed a mean reduction of −1.5 (95% CI, −1.8 to −1.1), while the UG-SNK-RE group showed a mean reduction of −2.5 (95% CI, −2.9 to −2.1). The between-group difference in VAS score was −1.0 (95% CI, −1.4 to −0.6; *P* < 0.001) in favor of the UG-SNK-RE group. At the 8-week follow-up, the UG-SNK-RE group showed a significant between-group difference of −1.1 (95% CI, −1.5 to −0.7; *P* < 0.001) compared with the NSAIDs-RE group (Table 2). A comparison of VAS and WOMAC scores between the two groups is presented in Figure 2.

**Table 2:** VAS scores among study participants.

Time	VAS score, mean (SD)		Mean change in VAS score from baseline (95% CI)		NSAIDs-RE group vs. UG-SNK-RE group		Group × time interaction	Time	Group
	NSAIDs-RE group (*n* = 40)	UG-SNK-RE group (*n* = 40)	NSAIDs-RE group	UG-SNK-RE group	Difference (95% CI)	*P* value			
4 week	3.7 (1.1)	2.7 (0.9)	−1.5 (−1.8 to −1.1)	−2.5 (−2.9 to −2.1)	−1.0 (−1.4 to −0.6)	<0.001	*F* = 14.7	*F* = 251.9	*F* = 20.4
Baseline	5.1 (1.0)	5.2 (1.0)	ON	ON	0.1 (−0.3 to 0.4)	0.8			
8 week	3.1 (0.8)	1.9 (0.6)	−2.1 (−2.5 to −1.6)	−3.3 (−3.7 to −2.8)	−1.1 (−1.5 to −0.7)	<0.001	*P*<0.001	*P*<0.001	*P*<0.001

Secondary outcomes are presented in Table 3. At the 4-week time point, the UG-SNK-RE group showed significant improvements in several secondary outcomes compared to the NSAIDs-RE group. These included WOMAC pain (−1.6 [−2.4 to −0.8], *P* < 0.001), WOMAC stiffness (−0.8 [−1.3 to −0.4], *P* < 0.001), WOMAC function (−3.1 [−4.8 to −1.4], *P* < 0.001), depth of patellar effusion (−0.8 [−1.2 to −0.5], *P* < 0.001), synovial thickness (−0.7 [−1.1 to −0.3], *P* < 0.001), vastus lateralis thickness (0.5 [0.1–0.9], *P* = 0.007), vastus medialis oblique thickness (1.6 [1.2–2.1], *P* < 0.001), and medial collateral ligament thickness (−0.3 [−0.6 to −0.1], *P* = 0.016). However, no significant differences were observed for the thickness of the rectus femoris (−0.1 [−0.5–0.7, *P* = 0.73) or the lateral collateral ligament (0.1 [−0.1–0.3], *P* = 0.44).

**Table 3:** Secondary outcomes at baseline, 4, and 8 weeks.

Outcome	Mean (SD)		Mean change from baseline (95% CI)		NSAIDs-RE group vs. UG-SNK-RE group		Group × time interaction	Time	Group
	NSAIDs-RE group (*n* = 40)	UG-SNK-RE group (*n* = 40)	NSAIDs-RE group	UG-SNK-RE group	Difference (95% CI)	*P* value			
WOMAC pain									
4 week	7.2 (1.9)	5.6 (1.4)	−2.8 (−3.3 to −2.2)	−4.5 (−5.0 to −3.9)	−1.6 (−2.4 to −0.8)	<0.001	F = 19.8	F = 332.1	F = 13.2
Baseline	10.0 (2.0)	10.1 (2.6)	ON	ON	0.8 (−0.7 to 0.9)	0.854			
8 week	6.5 (1.5)	4.2 (1.0)	−3.5 (−4.2 to −2.8)	−5.9 (−6.6 to −5.2)	−2.3 (−3.1 to −1.5)	<0.001	*P*<0.001	*P*<0.001	*P*<0.001
WOMAC stiffness									
4 week	3.8 (1.0)	3.0 (0.8)	−1.1 (−1.5 to −0.7)	−2.0 (−2.4 to −1.6)	−0.8 (−1.3 to −0.4)	<0.001	F = 10.7	F = 191.8	F = 12.7
Baseline	4.9 (1.2)	5.0 (1.3)	NA	NA	0.1 (−0.3 to 0.5)	0.7			
8 week	2.8 (0.9)	1.7 (0.6)	−2.1 (−2.6 to −1.6)	−3.3 (−3.7 to −2.8)	−1.1 (−1.5 to −0.6)	<0.001	*P*<0.001	*P*<0.001	*P* = 0.001
WOMAC function									
4 week	20.2 (4.4)	17.1 (3.0)	−5.0 (−6.0 to −4.0)	−8.0 (−8.9 to −7.0)	−3.1 (−4.8 to −1.4)	<0.001	F = 52.9	F = 344.3	F = 23.8
Baseline	25.2 (4.8)	25.1 (4.4)	NA	NA	−0.2 (−1.8 to 1.5)	0.9			
8 week	19.5 (3.7)	11.6 (2.0)	−5.8 (−7.1 to −4.4)	−13.5 (−14.8 to −12.2)	−7.9 (−9.6 to −6.2)	<0.001	*P*<0.001	*P*<0.001	*P*<0.001
Depth of patellar sac effusion,mm									
4 week	2.7 (0.7)	1.9 (0.7)	−0.8 (−1.1 to −0.5)	−1.7 (−2.0 to −1.4)	−0.8 (−1.2 to −0.5)	<0.001	F = 11.1	F = 171.1	F = 13.2
Baseline	3.6 (1.0)	3.6 (0.9)	NA	NA	0.1 (−0.3 to 0.4)	0.8			
8 week	1.9 (0.7)	1.3 (0.5)	−1.7 (−2.1 to −1.3)	−2.3 (−2.7 to −1.9)	−0.6 (−0.9 to −0.3)	<0.001	*P*<0.001	*P*<0.001	*P*<0.001
Thickness of synovial,mm									
4 week	2.4 (0.8)	1.7 (0.7)	−0.7 (−1.0 to −0.4)	−1.5 (−1.8 to −1.2)	−0.7 (−1.1 to −0.3)	<0.001	F = 10.7	F = 90.6	F = 11.7
Baseline	3.2 (1.2)	3.2 (1.1）	NA	NA	0.1 (−0.3 to 0.4)	0.8			
8 week	2.3 (0.6)	1.4 (0.5)	−0.9 (−1.3 to −0.5)	−1.9 (−2.2 to −1.5)	−0.9 (−1.3 to −0.5)	<0.001	*P*<0.001	*P*<0.001	*P* = 0.001
Thickness of vastus lateralis									
4 week	17.1 (0.7)	17.6 (0.6)	0.3 (−0.1 to 0.6)	0.6 (0.2 to 0.9)	0.5 (0.1 to 0.9)	0.007	F = 4.3	F = 19.8	F = 15.5
Baseline	16.8 (1.3)	17.0 (1.0)	NA	NA	0.2 (−0.2 to 0.6)	0.2			
8 week	17.2 (0.8)	18.2 (0.7)	0.4 (−0.001 to 0.9)	1.2 (0.7 to 1.6)	1.0 (0.6 to 1.3)	<0.001	*P* = 0.016	*P*<0.001	*P*<0.001
Thickness of vastus medialis oblique									
4 week	11.5 (1.0)	13.1 (0.7)	0.5 (0.04 to 0.9)	2.2 (1.7 to 2.6)	1.6 (1.2 to 2.1)	<0.001	F = 39.2	F = 118.1	F = 78.5
Baseline	11.0 (1.2)	10.9 (1.2)	NA	NA	−0.1 (−0.5 to 0.4)	0.8			
8 week	11.9 (0.8)	14.3 (0.7)	1.0 (0.5 to 1.4)	3.4 (2.9 to 3.9)	2.4 (2.0 to 2.8)	<0.001	*P*<0.001	*P*<0.001	*P*<0.001
Thickness of rectus femoris									
4 week	13.3 (1.0)	13.4 (1.4)	0.4 (−0.1 to 0.8)	0.3 (−0.1 to 0.7)	−0.1 (−0.5 to 0.7)	0.7	F = 0.1	F = 5.2	F = 0.4
Baseline	12.9 (1.5)	13.0 (1.6)	NA	NA	0.1 (−0.5 to 0.7)	0.7		*P* = 0.007	
8 week	13.3 (0.9)	13.5 (1.2)	0.4 (−0.1 to 0.9)	0.5 (−0.02 to 1.0)	0.2 (−0.4 to 0.8)	0.4	*P* = 0.868		*P* = 0.55
Thickness of medial collateral ligament									
4 week	5.3 (0.4)	4.9 (0.3)	−0.1 (−0.4 to 0.1)	−0.5 (−0.8 to −0.3)	−0.3 (−0.6 to −0.1)	0.016	F = 4.7	F = 17.4	F = 6.6
Baseline	5.4 (1.0)	5.4 (0.9)	NA	NA	0.1 (−0.2 to 0.3)	0.7			
8 week	5.1 (0.3)	4.6 (0.4)	−0.3 (−0.6 to 0.1)	−0.8 (−1.1 to −0.5)	−0.5 (−0.8 to −0.2)	=0.001	*P*=0.01	*P* < 0.001	*P*=0.12
Thickness of lateral collateral ligament									
4 week	4.8 (0.5)	4.9 (0.4)	−0.1 (−0.3 to 0.1)	−0.1 (−0.3 to 0.1)	0.1 (−0.1 to 0.3)	0.4	F = 0.1	F = 5.6	F = 0.8
Baseline	4.9 (0.7)	5.0 (0.5)	NA	NA	0.10 (−0.1 to 0.3)	0.4			
8 week	4.7 (0.4)	4.7 (0.5)	−0.2 (−0.5 to 0.02)	−0.3 (−0.5 to −0.02)	0.1 (−0.2 to 0.3）	0.6	*P* = 0.95	*P* = 0.004	*P* = 0.37

At the 8-week follow-up, the UG-SNK-RE group continued to show superior results in treating KOA, with significant improvements in WOMAC pain (−2.3 [−3.1 to −1.5], *P* < 0.001), WOMAC stiffness (−1.1 [−1.5 to −0.6], *P* < 0.001), WOMAC function (−7.9 [−9.6 to −6.2], *P* < 0.001), depth of patellar effusion (−0.6 [−0.9 to −0.3], *P* < 0.001), synovial thickness (−0.9 [−1.3 to −0.5], *P* < 0.001), vastus lateralis thickness (1.0 [0.6–1.3], *P* < 0.001), vastus medialis oblique thickness (2.4 [2.0–2.8], *P* < 0.001), and medial collateral ligament thickness (−0.5 [−0.8 to −0.2], *P* = 0.001). No significant differences were found for rectus femoris thickness (0.2 [−3.5–0.8], *P* = 0.44) or lateral collateral ligament thickness (0.05 [−0.2–0.3], *P* = 0.66). Within the UG-SNK-RE group, significant changes were observed across all time points (baseline, 4-week, and 8-week). Importantly, no adverse events were reported by any participant throughout the study.

## Discussion

KOA is a chronic degenerative bone disease that leads to joint pain, stiffness, functional decline, and, in severe cases, disability (47). With the aging population, the incidence of KOA has been increasing annually (48). Although there has been considerable progress in understanding the mechanisms of KOA, no interventions have been approved to slow or prevent its progression, and current treatments primarily focus on symptom management (49–51). In China, complementary and alternative therapies for degenerative bone diseases have shown promising results, which may explain the high level of participant compliance in this clinical trial (52, 53).

Compared to the NSAIDs-RE group, the UG-SNK-RE group demonstrated a significantly greater reduction in VAS scores at the end of the 4-week intervention, with a sustained decrease in pain intensity through the 8-week follow-up. Although the NSAIDs-RE group also showed a statistically significant reduction in VAS scores from baseline at the end of the intervention (−1.45 [−1.81 to −1.09], *P* < 0.001), a decrease of at least 2 points on the VAS scale was considered clinically meaningful. At both 4-week and 8-week time points, participants in the UG-SNK-RE group exhibited significant improvements in WOMAC scores for pain, stiffness, and function, as well as reductions in the depth of patellar sac effusion, synovial thickness, and the thickness of muscles (vastus lateralis and vastus medialis oblique), as well as the medial collateral ligament.

The small needle knife therapy has been widely used in China for treating KOA, with notable success in reducing knee joint pain and improving mobility. Consistent with these findings, our study demonstrated that ultrasound-guided small needle knife therapy combined with resistance exercises effectively alleviates pain and enhances joint mobility. A similar study (22) reported that small needle knife therapy led to a significantly greater improvement in the Oxford Knee Score compared to celecoxib administration, although no significant differences were found in gait performance. Notably, some participants in that study withdrew due to safety concerns (e.g., bleeding), a common side effect of traditional small needle knife surgery (7). In contrast, our study utilized ultrasound guidance to minimize the risk of vascular and nerve damage, resulting in no adverse events. To our knowledge, this is the first randomized controlled trial to demonstrate the efficacy and safety of ultrasound-guided small needle knife therapy combined with resistance exercise for patients with KOA.

Recent research has highlighted the loss of lower limb muscle mass and strength as an independent risk factor for osteoarthritis (OA) (54, 55). Irfan et al. found a significant negative correlation between quadriceps thickness and the duration, stage, and severity of symptoms in patients with KOA (56). Additionally, muscle mass may influence both the symptoms (e.g., pain) and treatment outcomes in OA (57). Exercise therapy is recommended as a first-line treatment for enhancing muscle strength and restoring joint function in KOA patients (58, 59). Resistance exercise, in particular, has proven to be both safe and effective in improving muscle strength and quality in these patients (60, 61). For instance, one study showed that high-intensity water resistance training significantly improved walking speed in women with mild KOA (62). However, several studies have since reported no significant differences in walking speed or muscle strength between high- and low-intensity resistance training in KOA patients. In line with these findings, our study demonstrated that ultrasound-guided small needle knife therapy combined with resistance exercises can significantly improve muscle and ligament function, while also alleviating clinical symptoms. Ultrasound guidance minimizes potential adverse effects of needle knife surgery, such as bleeding and nerve damage, and enables real-time assessment of tissue changes (muscles and ligaments) before and after treatment. This rigorously designed trial, led by experienced needle knife practitioners and incorporating carefully tailored resistance exercises, provides valuable clinical evidence supporting ultrasound-guided small needle knife therapy as an alternative treatment for KOA.

Clinically, this combined therapeutic strategy can be clearly positioned in the standardized KOA management sequence as a safe and effective minimally invasive complementary regimen for middle and early-stage KOA patients. Specifically, patients eligible for this approach are those with grade I–II KOA according to the Kellgren-Lawrence classification, who present with persistent mild-to-moderate knee pain, joint stiffness, synovial hyperplasia, effusion, and decreased periarticular muscle strength, and fail to achieve satisfactory and sustained symptomatic relief after routine first-line conservative treatments including oral NSAIDs, conventional physical therapy, and single exercise intervention. Meanwhile, this treatment is also suitable for patients who cannot tolerate the long-term side effects of nonsteroidal anti-inflammatory drugs or have poor adherence to single physical and exercise rehabilitation. For advanced KOA patients with severe irreversible joint cartilage destruction, bony hyperplasia and joint deformity, or patients requiring surgical intervention, this combined therapy can only serve as an adjuvant palliative treatment rather than a dominant therapeutic option. This hierarchical clinical application scope enables clinicians to precisely screen eligible patients and standardize the clinical application of ultrasound-guided small needle knife combined with resistance exercise in KOA management.

### Limitations

This study has several limitations. First, due to the nature of the needle knife intervention, both physicians and participants were unable to be blinded, which may have introduced performance bias. Second, the study's single-center design and relatively small sample size may limit the generalizability of the findings. Third, monitoring participants’ adherence to daily resistance exercises posed a challenge. Despite strict guidelines and regular follow-ups through WeChat, it was difficult to ensure full compliance with the prescribed exercises. Fourth, the possibility of participant expectation bias cannot be ruled out, which may have led to an overestimation of benefits. However, the fact that some objective ultrasound indicators did not show significant improvement suggests that this bias may be minimal. Finally, the study's follow-up period was limited to 8 weeks, including baseline, 4 weeks of treatment, and 4 weeks of follow-up. Longer–term outcomes could provide more clinically relevant data and should be explored in future studies.

## Conclusion

In this randomized clinical trial, patients with knee osteoarthritis who received ultrasound-guided small needle knife therapy combined with resistance exercise experienced greater improvements in pain intensity, joint function, and muscle and ligament health compared to those receiving nonsteroidal anti-inflammatory drugs combined with resistance exercise. These findings suggest that ultrasound-guided small needle knife therapy, when used alongside resistance exercises, could be an effective supplementary treatment for managing knee osteoarthritis.

## Data Availability

The original contributions presented in the study are included in the article/Supplementary Material, further inquiries can be directed to the corresponding authors.

## Supplementary material

Supplementary Material 1 Full trial protocol of this randomized clinical trial.

Supplementary Material 2 Detailed resistance-exercise intervention protocol.
