# Supplementary material for: Effect of ultrasound-guided small needle knife on patients with knee osteoarthritis: a randomized clinical trial
Source: Front Med (Lausanne). 2026 Sep 4;13:1902456. doi: 10.3389/fmed.2026.1902456 (PMC13585640; doi:10.3389/fmed.2026.1902456)
Supplement: Supplementary file 1 [file Supplementaryfile1.doc]

**Supplementary Online Content**

**1.Resistance exercise protocol**

Before enrollment, both groups of participants received 2 hours of health education to improve compliance. All participants in this study were instructed to perform necessary resistance exercises. Prior to exercising, participants were asked to warm up for 5 minutes. The specific resistance exercises included:

**Supine Resistance Straight Leg Raise**: Participants lay on their back with a 2 kg sandbag attached to the ankle of the affected leg. They extended the leg and raised it to a 30° angle relative to the bed, holding for 10 seconds. This exercise was repeated 20 times per set.

**Lateral Resistance Straight Leg Raise:** Participants lay on their side with a 2 kg sandbag on the affected leg's ankle. The leg was extended and raised to a 30° angle relative to the bed, held for 10 seconds, and repeated for 20 repetitions per set.

**Sitting Resistance Straight Leg Raise:** In a seated position, participants fixed a 2 kg sandbag at the ankle of the affected leg. The knee joint was gradually extended until the leg formed a 90° angle with the body, holding for 10 seconds. The exercise was repeated 20 times per set.

**Standing Resistance Knee Bend:** While standing, participants fixed a 2 kg sandbag at the ankle of the affected leg. They then bent the knee so that the calf and thigh formed a 90° angle, holding for 10 seconds. This exercise was repeated for 20 repetitions per set.

During the 4-week intervention, participants were instructed to perform the resistance exercises at least twice daily, with the option to practice at home. Participants were asked to record their exercises and send the videos to the physician via email or WeChat. The physician reviewed the videos and provided feedback on proper technique and suggestions for improvement.

**Fig1. The main movements of Resistance exercise**. A: Supine resistance straight leg raising. B: Lateral resistance straight leg raising. C: Sitting resistance straight leg raising. D: Standing resistance knee bending exercise.

2. **Ultrasound guided small needle knife operation**

Participants in the ultrasound-guided small needle knife combined with resistance exercise group will receive small needle knife therapy under ultrasound guidance. The procedure will be performed as follows: 1. Positioning: The participant lies in a supine position with the affected limb extended. A thin pillow is placed under the affected knee to facilitate flexion. 2. Preoperative Preparation: The skin area extending 20 cm above and below the affected patella is disinfected three times. The physician wears sterile gloves, and the ultrasound probe, coated with a sterile coupling agent, is covered with a sterile plastic sleeve. Sterile coupling agent is applied between the probe and the skin. 3. Identification of Fixed Points: Based on the "Practical Clinical Practice of Small Needle Knife" and the "Guidelines for Traditional Chinese Medicine Diagnosis and Treatment of Knee Osteoarthritis (2020 Edition)", the needle knife treatment targets the internal and external patellar retinaculum, the starting and ending points of the internal and external collateral ligaments, and the termination points of the quadriceps tendon. Tender points are identified using the "pain as a point" principle and marked with gentian violet. 4. Ultrasound-Guided Needle Knife Procedure: A 10 mL injection of 2% lidocaine is administered for local anesthesia. The ultrasound probe scans the marked points to identify the precise locations. Using a small needle knife held at a 45° angle to the skin, the needle is inserted parallel to the tissue’s long axis. Three longitudinal cuts are made, followed by two transverse cuts after rotating the needle knife 90°. The needle knife is then advanced to the bone surface to perform periosteal cutting. The procedure is performed at each marked point. Afterward, the area is compressed for 3–5 minutes to control bleeding, disinfected, and covered with a sterile dressing. The procedure is repeated weekly, with a total of 4 weeks constituting one course of treatment.

**Fig2. Schematic diagram of needle knife operation under ultrasound guidance.** A: Participant position. B:Ultrasound guided needle knife operation site diagram. C:Needle knife operation under ultrasound of quadriceps tendon(PA: Patella; RF: Rectus femoris). D:Needle knife operation under patellar ligament ultrasound(TT: Tibial tuberosity; PL:Patellar ligament). E: Ultrasound guided needle knife operation with medial patellar support band(F: Femur; MeR: Medial Retinaculum). F:Needle knife operation guided by ultrasound at the proximal end of the medial collateral ligament(F: Femur; T:Tibia; MM:Medial meniscus; MCL:Medial collateral ligament). G:Needle knife operation guided by ultrasound at the distal end of the medial collateral ligament(T:Tibia; MCL:Medial collateral ligament). H:Ultrasound guided needle knife operation with lateral patellar support band(F: Femur; LR:lateral retinaculum). I:Needle knife operation guided by ultrasound at the proximal end of the lateral collateral ligament(LCL:Lateral collateral ligament; F: Femur). J:Needle knife operation guided by ultrasound at the distal end of the lateral collateral ligament(FH:fibular head; LCL:Lateral collateral ligament). The white triangle refers to the needle knife imaging under ultrasound.
